# Antioxidant enzyme profile and lipid peroxidation products in semen samples of testicular germ cell tumor patients submitted to orchiectomy

**DOI:** 10.1590/S1677-5538.IBJU.2016.0323

**Published:** 2017

**Authors:** Camila Sposito, Mariana Camargo, Danielle Spinola Tibaldi, Valéria Barradas, Agnaldo Pereira Cedenho, Marcílio Nichi, Ricardo Pimenta Bertolla, Deborah Montagnini Spaine

**Affiliations:** 1 Departamento de Cirurgia, Divisão de Urologia, Setor de Reprodução Humana, Universidade Federal de São Paulo, SP, Brasil;; 2 Departamento de Reprodução Animal, Faculdade de Medicina Veterinária, Universidade de São Paulo, SP, Brasil

**Keywords:** Antioxidants, Oxidative Stress, Testicular Germ Cell Tumor, Orchiectomy, Semen

## Abstract

**Purpose:**

To determine enzymatic antioxidant and lipid peroxidation levels in seminal plasma of patients orchiectomized for testicular tumors.

**Materials and Methods:**

The study included 52 patients: 26 control men and 26 orchiectomized patients for testicular tumor, of which 12 men had seminoma tumor and 14 men non-seminoma tumor. After semen analysis performed according to the WHO guidelines, an aliquot of semen was centrifuged and the seminal plasma was collected. Lipid peroxidation was performed by thiobarbituric acid reactive substances (TBARS) assay and antioxidant profile was assessed by analyzing catalase, glutathione peroxidase (GPx) and superoxide anion (SOD) activities using colorimetric assays with a standard spectrophotometer. Data were tested for normality and compared using one-way ANOVA (p<0.05).

**Results:**

Seminoma and non-seminoma groups presented lower sperm concentration and morphology when compared to control group (p=0.0001). Both study groups (seminoma and non-seminoma) presented higher TBARS levels when compared to control group (p=0.0000013). No differences were observed for SOD (p=0.646) andGPx (p=0.328). It was not possible to access the enzymatic activity of catalase in any group.

**Conclusion:**

Patients with testicular tumor present increased semen oxidative stress, but no differences were observed in antioxidant levels, even after orchiectomy. This indicates that most likely an increased generation of oxidative products takes place in these patients.

## INTRODUCTION

Testicular tumors account for about 1 to 2% of all kinds of tumors that affect men, causing approximately 0.1% of deaths from cancers in men (1-3). In Brazil, in 2013, the National Institute of Cancer (INCA) estimated approximately 343 deaths caused by testicular cancer (4).

Histologically, testicular tumors are divided into germ cell (95% of cases) and non-germ cell tumors (5% of cases) (5). Germ cell tumors are further divided into seminoma (60% of cases) and non-seminoma (40% of cases) (5-7). Seminomas are more frequent in men about 40 years old, while non-seminomas are more frequent in men between 20-35 years old (5, 8, 9).

The treatment for patients with a testicular tumor is orchiectomy of the affected testis via inguinal surgery, followed, in most cases, by adjuvant treatments such as chemotherapy, and/or radiotherapy, and/or retroperitoneal lymphadenectomy (10, 11). Although unilateral orchiectomy does not result directly in infertility, because the remaining testicle may present normal spermatogenesis, potential side effects are disruption of retroperitoneal sympathetic nerves, which may result in retrograde ejaculation (12), and a high risk of hypogonadotropic hypogonadismdue to the reduction in testosterone production (13).

Regardless of their classification, studies have reported the association of testicular tumors with testicular oxidative imbalance (14, 15) which can compromise sperm motility, concentration, and morphology, as well as interfere with sperm capacitation and fertilization (16-18). The seminal plasma, however, represents the main protection system, which includes a chain of enzymatic antioxidants, composed by superoxide dismutase (SOD), catalase (CAT) and glutathione peroxidase (GPx), which are capable of neutralizing ROS, thus preventing damage caused by oxidative stress (19).

Although it is well known that testicular tumors are harmful to the sperm membrane (20), it still remains to be clarified if orchiectomized patients return to a normal state after the surgery, or if oxidative stress is still present. Therefore, this study set out to investigate the enzymatic antioxidant profile and lipid peroxidation products in seminal samples of patients with testicular germ cell tumors after orchiectomy.

## MATERIALS AND METHODS

### Study design and patients

A cross-sectional study was performed with 52 men between 18-45 years old. The study groups comprised patients referred for semen cryopreservation at the Cell and Germinative Tissue Bank in the Federal University of São Paulo. The study groups included 12 patients orchiectomized for a unilateral seminoma testicular germ cell tumor (study group S) and 14 patients orchiectomized for a unilateral non-seminoma testicular germ cell tumor (study group NS), which did not present post-operative complications after the surgery. The semen collection was realized one month after surgery, before the beginning of any adjuvant therapy. The control group was composed of 26 healthy men without testicular germ cell tumor and semen quality within the 95% fertile range of the 2010 World Health Organization (WHO) guidelines (21), seeking the Human Reproduction Sector, Division of Urology, Sao Paulo Federal University Andrology Laboratory for semen analysis. Patients referring fever in the 90 days period prior to semen analysis, with evidence of urogenital infection, and patients with a history of cancer or endocrinophaties (and their treatments), smokers, individuals with BMI ≥30 and individuals who had not yet been submitted to orchiectomy were excluded from the study. Institutional Review Board approval was obtained from the Sao Paulo Federal University Research Ethics Committee.

### Semen analysis

Semen samples were obtained by masturbation after 2 to 5 days of ejaculatory abstinence. After liquefaction, semen analysis was conducted according to the WHO guidelines (21). Immediately after the semen analysis, 0.5mL was centrifuged at 16.000*g* for 1h at 4ºC to remove all the cellular debris. The supernatant seminal plasma was then collected and stored at -20ºC until the time of the analyses. All of the remaining semen volume was transported to the Germ Cell and Tissue Bank of the Human Reproduction Sector / Division of Urology of the Sao Paulo Federal University for cryopreservation (22).

### Lipid Peroxidation assessment

Lipid peroxidation was determined with the method described by Ohkawa et al. (23), which is based on the determination of its products, mainly the malondialdehyde (MDA) levels, due to its reaction with thiobarbituric acid (TBA). To precipitate proteins, 500µL of seminal plasma and 1000µL of a 10% solution (v:v) of trichloroacetic acid (TCA 10%) were mixed and centrifuged (18.000 x g for 15 min at 15ºC). After centrifugation, 500µL of the supernatant and 500µL of 1% (v:v) thiobarbituric acid (TBA, 1%), in 0.05N sodium hydroxide in glass tubes were placed into a boiling water bath (100ºC) for 10 min, and subsequently cooled in an ice bath (0ºC) to stop the chemical reaction. The TBARS were then quantified using a spectrophotometer (UV-vis Spectrophotometer Ultrospec 3300 Pro; BiochromLtd., Cambridge, UK) at a wave length of 532nm. The results were compared with a previously prepared standard curve with a standard solution of malondialdehyde (Sigma-10.838-3-St Louis, USA). Lipid peroxidation levels were described in nanograms of TBARS/mL of seminal plasma. TBARS levels were then normalized to sperm concentration (TBARS/sperm).

### Antioxidant seminal profile

Catalase activity was determined indirectly through monitoring hydrogen peroxide consumption (H_2_O_2_). The reaction solution contained 10µL of seminal plasma added to 90µL of Tris(hydroxymethyl)-amino-methane/EDTA buffer solution (50 and 250mM, respectively) and 900µL of H_2_O_2_ (9.0mM). The reaction was allowed to take place at pH 8.0, 30ºC, for 8 min, and then the enzymatic activity was measured using a spectrophotometer (wavelength, 230nm). The absorbance was measured every 5 seconds, generating a curve of H_2_O_2_ consumption that was compared with a blank sample. The calculations considered the value 0.071M-1 x cm-1 as a molar extinction coefficient of H_2_O_2_. The activity of the enzyme catalase was calculated based on the formula [catalase activity=(initial absorbance-final absorbance)/0.071 x dilution] and the result expressed in UI/mL (24).

The enzymatic activity of the Glutathione peroxidase(GPx) was determined indirectly by measuring the consumption of reduced nicotinamide adenine dinucleotide phosphate (NADPH) (24). The assay mixture consisted of NADPH (0.12mM, 1mL), GSH (1mM, 100mL), GSSGr (0.25U/mL, 20mL), and sodium azide (0.25mM, 20mL). A volume of 100µL of seminal plasma was used. The spectrophotometer cell was brought up to a volume of 1.9mL with phosphate buffer 143mM, EDTA 6.3mM, (pH 7.5), that was also used to dissolve the NADPH. The GSH was dissolved in 5% metaphosphoric acid. Sodium azide was used to inhibit the action of catalase. This reaction was initiated with the addition of 1.2mM of tert-butyl hydro peroxide (TBHP, 100mL), and the consumption of NADPH was detected at a wavelength of 340nm, for 10 min at 37^o^C (measurements performed every 5 seconds). The results of GSH-Px were expressed as units of GSH-Px/mL of semen, and calculations used 6.22mM-1 x cm-1 as the extinction coefficient of NADPH (24).

The SOD activity was measured indirectly by means of the reduction rate of cytochrome C by superoxide anion (O_2_-) controlled every 5 min. The xanthine-xanthine oxidase system was used as a continuous generated of O_2_- that, in turn, causes the reduction of cytochrome C. The total SOD in seminal sample competes with cytochrome C and superoxide anion (O_2_-). The total SOD activity was measured indirectly by determining the decrease rate in the reduction of cytochrome C (Floré & Otting, 1984). The assay mixture consisted of 10µL of seminal plasma, 835µL of a solution containing cytochrome C (1mM) and xanthine (50mM), and 155µL of xanthine oxidase diluted in sodium phosphate/EDTA buffer (50 and 100mM, respectively, pH 7.8). The concentration of xanthine oxidase was calculated to generate the optimum amount of O_2_ with a consequent reduction of cytochrome C that was calculated as the rate of cytochrome C reduction of 0.025 units of absorbance/min (at 550nm of wavelength); the basis of this calculation is that 1 unit of total SOD activity corresponded to 50% of this value. Therefore, SOD activity in the sample decreased the rate of cytochrome reduction when compared to the blank.

### Statistical analysis

The data were analyzed in the SAS System for Windowssoftware (SAS, 2000). The applicative Guided Data Analysis was used to test the data for residue normality (normal distribution) and homogeneity of variances. If normality was not observed, variables were transformed into their logarithmic or square root values. If after transformation normality was not observed, a non-parametric analysis was carried out using theNPAR1WAY procedure. For data with normal distribution, the ANOVA test followed bya Least Significant Differences (LSD) post-hoc test was used to compare the groups. An α of 5% was considered for all the analyses.

## RESULTS

Individual characteristics and semen quality results are presented in Table-1. No statistically significant differences were observed in age, ejaculate volume, sperm motility, and round cells and neutrophil concentration between the three groups. The seminoma and non-seminoma presented lower sperm concentration, morphology, and total sperm count when compared to the control group.

**Table 1:** Semen analysis of orquiectomized men and healthy control men. Groups were compared by ANOVA followed by LSD post-hoc test.

	Control Group (n= 26)	Seminoma Group (n= 12)	Non-Seminona Group (n= 14)	p
**Age (years)**				
Mean; SD	29.8; 3.27	28.4; 6.02	26.7; 6.17	0.165
95% CI	[28.56; 31.21]	[24.59; 32.24]	[23.22; 30.35]	
**Abstinence (days)**				
Mean; SD	4.4; 3.47	6.7; 7.44	5.3; 6.00	0.993
95% CI	[3.06; 5.87]	[2.02; 11.48]	[1.89; 8.83]	
**Volume (mL)**				
Mean; SD	3.6; 0.97	3.8; 1.73	3.2; 1.30	0.478
95% CI	[3.23; 4.02]	[2.76; 4.96]	[2.45; 4.02]	
**Progressive motility (%)**				
Mean; SD	56.6; 7.05	63.1; 6.43	58.2; 9.80	0.098
95% CI	[53.84; 59.54]	[58.49; 67.70]	[52.63; 63.94]	
**Non-progressive motility (%)**				
Mean; SD	4.5; 1.98	3.8; 2.35	5.0; 2.09	0.340
95% CI	[3.70; 5.30]	[2.02; 5.40]	[3.86; 6.28]	
**Sperm concentration (X10** ^**6**^ **/mL)**				
Mean; SD	82.4; 53.48^b^	18.1; 23.06^a^	16.7; 17.26^a^	<0.0001*
95% CI	[60.82; 104.02]	[2.69; 33.68]	[6.79; 26.73]	
**Total sperm count (X10** ^**6**^ **)**				
Mean; SD	295.7; 213.03^b^	59.6; 72.82^a^	69.6; 85.70^a^	<0.0001*
95% CI	[209.73; 381.82]	[13.36; 105.89]	[20.14; 119.11]	
**Morphology (% normal)**				
Mean; SD	14.4; 0.85^b^	5.0; 3.22^a^	5.4; 2.06^a^	<0.0001*
95% CI	[14.11; 14.81]	[2.97; 7.06]	[4.24; 6.62]	
**Round cells (X10** ^**6**^ **/mL)**				
Mean; SD	0.9; 0.74	0.7; 1.08	1.3; 0.97	0.304
95% CI	[0.68; 1.28]	[0.07; 1.45]	[0.74; 1.86]	
**Neutrophils (X10** ^**6**^ **/mL)**				
Mean; SD	0.1; 0.17	0.1; 0.18	0.2; 0.50	0.577
95% CI	[0.04; 0.18]	[-0.02; 0.22]	[-0.09; 0.50]	

**SD=**Standard deviation

**95% CI =**Confidence interval of 95% of the mean

* – significant difference

Different Letters in the same row indicate significant difference (post-hoc LSD test – p<0.05).

The variables TBARS, GPx, SOD and TBARS/sperm of the three groups (seminoma, non-seminoma, and control) are shown in Table-2. The seminoma and non-seminoma groups presented higher TBARS levels when compared to controls. No statistically significant differences were observed for SOD and GPx activities. No reading levels were achieved for catalase enzyme activity by the Beutler method in any group.

**Table 2:** TBARS, Glutathione Peroxidase (GPx) and Superoxide Dismutase (SOD) levels from orquiectomized men and healthy control men. Groups were compared by ANOVA followed by LSD post-hoc test.

	Control Group (n= 26)	Seminoma Group (n=12)	Non-seminoma Group (n=14)	p
**TBARS/sperm**				
Mean; SD	3.2; 2.59^b^	53.2; 127.19^a^	22.7; 25.95^a^	<0.001*
95% CI	[2.16; 4.26]	[-27.62; 134.01]	[7.74; 37.71]	
**GPx (UI/mL)**				
Mean; SD	65.0; 20.02	59.9; 19.45	67.5; 23.05	0.646
95% CI	[56.97; 73.15]	[47.63; 72.35]	[54.21; 80.83]	
**SOD (UI/mL)**				
Mean; SD	63.9; 59.84	34.3; 30.64	50.0; 27.49	0.328
95% CI	[39.79; 88.13]	[14.88; 53.83]	[34.16; 65.92]	

**SD=**Standard deviation

**95% CI=**Confidence interval of 95% of the mean

* – significant difference

Different Letters in the same row indicate significant difference (post-hoc LSD test – p<0.05).

## DISCUSSION

Whichever the histological origin of the testicular germ cell tumor (seminoma or non-seminoma), orchiectomy is the initial therapeutic approach, usually followed by gonadotoxic adjuvant therapies, such as chemotherapy and radiotherapy (25). However, even before any treatment for the cancer begins, patients may display reduced fertility potential because of alterations to the testicular environment due to the disease itself (14, 15).

To our knowledge, there is no study in the literature that evaluates the role of oxidative stress and antioxidants in the seminal plasma of testicular germ cell tumor patients after orchiectomy. Thus, this study aimed to verify the activity of the main seminal antioxidant enzymes SOD, GPx, and catalase as well as the oxidative by-product malondialdehyde. The seminal non-enzymatic antioxidant milieu consists of GPx, SOD and catalase to physiologically control the balance between ROS production and neutralization (26). In this study, catalase levels were undetected. However, determining catalase activity in seminal plasma remains a matter of debate, because the presence of this antioxidant in the semen is usually due mainly to the presence of neutrophils (24).Because the semen of our patients presented low concentration of these cells (under 0.5million/mL in either group), this could explain why catalase was not detected in this study.

GPx protects sperm membrane from oxidative stress and is involved in redox regulation (27). In addition, SOD is responsible for dismutation of superoxide radicals (O_2_-)into H_2_O_2_ and O_2_ (28). Regarding the seminoma and non-seminoma groups,when compared to healthy control men, no differences were observed in the SOD and GPx activity, suggesting that antioxidant mechanisms were not altered in the presence of a testicular germ cell tumor followed by orchiectomy. This may stem from the fact that most of the seminal antioxidant levels is supported by secretions from the prostate and seminal vesicles (27, 28), which are apparently unaffected by the presence of a testicular cancer, at least in terms of their production of enzymatic antioxidants. It could be argued that epididymal antioxidants also contribute to the seminal antioxidant capacity, but their contribution is generally described as much lower (29), and it is still not known whether epididymal secretion of antioxidants is affected by the tumor.

However, even with unchanged levels of antioxidants, sperm from patients of the seminoma and non-seminoma groups showed greatly increased sensibility to oxidative damage - demonstrated by increased TBARS levels normalized to sperm concentration -when compared to controls. Besides the mean looks different by the observer, this study did not observe statistical difference in TBARS/sperm of seminoma vs. non-seminoma patients.The cancer itself causes an increase in ROS levels (30), which may be mainly due to: (i) increased metabolic activity and energy produced by mitochondria (30) and (ii) chronic inflammation and cytokine releasing (31).Moreover, the presence of a testicular cancer may produce an inflammatory state in the contralateral testis (31), which in turn may lead to delayed spermatogenesis, thus increasing the production of immature sperm (20). This sperm-centered oxidative stress (as demonstrated by our results) would explain why lipid peroxidation levels would depend on sperm concentration - the latter acting as a substrate for the former (32). Given that,in our study, testicular germ cell tumor patients collected semen samples on average one month after orchiectomy,the inflammatory state would not have had time to be fully resolved, as one full cycle of spermatogenesis would not have yet occurred. This is further supported by the fact that the study group presented decreased sperm concentration (quantity) and morphology (quality).The findings corroborate with Tavilani et al. (33) study, which observed antioxidant profile and oxidative stress in asthenozoospermic men and only observed a significant difference in TBARS/sperm, indicating that the mechanism of oxidative stress occurs similarly in infertile men and testicular germ-cell tumor patients.

Besides, this study is limited to the fact that the patients have a short period to perform a sample collection, because after they are submitted to orchiectomy, the adjuvant therapy is required. This way, it is not possible to observe antioxidant profile difference after a complete spermatogenesis cycle. In addition, the number of patients included in the study could not be enough to demonstrate difference in some studied parameters. Moreover, a further path for future research would be increase the number of patients and include pre-operative samples, which would add information regarding how the antioxidants and lipid peroxidation act in a tumor milieu.

## CONCLUSIONS

In short, we verified that there is an imbalance on lipid peroxidation in patients with testicular germ cell tumors when compared with healthy individuals. Therefore, patients with seminoma and non-seminoma tumors demonstrated an increased seminal oxidative stress. Also, there was no difference in antioxidant levels, after orchiectomy. This seminal increase in oxidative stress is attributed to increased ROS generation caused by the tumor itself. In conclusion, orchiectomized patients, when compared to healthy controls, do not differ in terms of enzymatic antioxidant levels, but present lower semen quality and increased sperm-centered oxidative stress.
